# Soil maltase activity by a glucose oxidase–perioxidase system

**DOI:** 10.1007/s13205-012-0050-z

**Published:** 2012-02-25

**Authors:** Priscilla M. Mfombep, Zachary N. Senwo

**Affiliations:** 1 Department of Agronomy, Kansas State University, Manhattan, KS 66506 USA; 2 Department of Biological and Environmental Sciences, College of Agricultural, Life and Natural Sciences, Alabama A&M University, P.O. Box 1087, Normal, AL 35762 USA

**Keywords:** Soil maltase activity, Maltose, Glucose oxidase–peroxidase system, Carbon cycling

## Abstract

The enzyme maltase (glucoinvertase; glucosidosucrase; maltase-glucoamylase; α-glucopyranosidase; glucosidoinvertase; α-d-glucosidase; α-glucoside hydrolase; α-1,4-glucosidase EC 3.2.1.20), is involved in the exo-hydrolysis of 1,4-α-glucosidic linkages and certain oligosaccharides into glucose which is an important energy source for soil microbes. This enzyme originates from different sources, which include plants, seaweeds, protozoa, fungi, bacteria, vertebrates, and invertebrates. The assay of soil maltase using maltose as substrate and the released glucose determined using a glucose oxidase–peroxidase system has not been explored or investigated to the best of our knowledge. A simple assay protocol using this system is proposed to evaluate and characterize maltase activity in soils. The protocol involves the release of glucose (determined using a glucose oxidase–peroxidase colorimetric approach) when 1 g soil is treated with toluene and incubated with 5 mM maltose in 67 mM sodium acetate buffer (pH 5.0) at 37 °C for 1 h. The optimal activity using this procedure was at pH 5.0 and decreased at temperatures above 70 °C. The calculated *K*_m_ values ranged from 0.8 to 6.5 mM, and are comparable to those of enzymes purified from microorganisms. The Arrhenius equation plots for the activity in the four soils were linear between 20 and 70 °C. The activation energy values ranged from 34.1 to 57.2 kJ mol^−1^, the temperature coefficients (*Q*_10_) ranged from 1.5 to 1.9 (avg. = 1.7), and the coefficients of variation (CV) of the proposed assay protocol for the soils used was <6%. While we recognize the availability of established assay protocols to determine soil α-glucosidase (referred in other literature as maltase) activity based on the *p*-nitrophenol (artificial product) released from *p*-nitrophenyl-α-d-glucopyranoside (artificial substrate), our interest was to assay its activity by determining the glucose (natural product) released from maltose (natural substrate).

## Introduction

Maltase (glucoinvertase; glucosidosucrase; maltase-glucoamylase; α-glucopyranosidase; glucosidoinvertase; α-d-glucosidase; α-glucoside hydrolase; α-1,4-glucosidase EC 3.2.1.20), is involved in the exo-hydrolysis of 1,4-α-glucosidic linkages and certain oligosaccharides into glucose (Saha and Zeikus 1991). The existence of maltase was initially demonstrated in 1880 (Wang and Hartman 1976). Since then, it has been reported to be widely distributed in nature and its activity detected in various plant extracts, seaweeds, protozoa, fungi, bacteria, vertebrates and invertebrates (Bahl and Agrawal 1972; Hutson and Manners 1965) and various bacteria, mammalian tissues, yeasts and molds (Yamasaki and Suzuki 1974; Wang and Hartman 1976). The importance of maltase in biological systems and its potential involvement in C cycling has long been recognized. The substrates for this enzyme include maltose and maltotriose (McWethy and Hartman 1979; Saha and Zeikus 1991; Wang and Hartman 1976), *p*-nitrophenyl α-d-glucopyranoside and 4-methylumbelliferyl α-d-glucopyranoside (Berthelot and Delmotte 1999).

Maltase activity in soils can be quantified by estimating the glucose resulting from maltose hydrolysis. Anthon and Barrett (2002) developed the 3-methyl-2-benzothiazolinonehydrozone (MBTH) to quantify reducing sugars in pure systems. This method is subject to interferences from proteinaceous and acid-insoluble materials commonly present in soils. Other methods described to measure reducing sugars in soils include the use of glucose oxidase–peroxidase system (Benefield 1971; Kapustka et al. 1981), Somogyi–Nelson procedure (Deng and Tabatabai 1994) and hexokinase–glucose-6-phosphate dehydrogenase enzymatic assay (Frey et al. 1999).

The assay of soil maltase using maltose as substrate and the released glucose determined using a glucose oxidase–peroxidase system has not been explored or investigated to the best of our knowledge. Preliminary experiments in our laboratory suggest that liberated glucose, when soil is incubated for 1 h with maltose monohydrate, can be determined quantitatively using a glucose oxidase–peroxidase colorimetric method. Maltose is a low molecular weight compound and the least common disaccharide in nature. It results from the incomplete hydrolysis of starch and is readily hydrolyzed by maltase to release d-glucose. The method which seems easy, specific, and efficient, involves the determination of glucose released when soil is incubated with maltose concentration ranging from 5 to 8 mM in sodium acetate buffer (67 mM, pH 5.0) and toluene (advisable to use a micro-filter and eliminate the use of toluene) at 37 °C for 1 h. The glucose released is quantified using a commercially available glucose oxidase–peroxidase reagent kit, specific and sensitive for glucose quantification. Thus, the overall objective of this study was to propose an assay protocol to determine soil maltase activity using maltose as the substrate and measuring the amount of glucose released by a glucose oxidase–peroxidase colorimetric approach. The enzymatic activity was characterized with respect to kinetic parameters (*K*_m_ and *V*_max_), activation energy *E*_a_, and temperature coefficient (*Q*_10_).

## Materials and methods

### Soils

The soils used (Table 1) were surface samples (0–15 cm) obtained from Alabama (Decatur, Houston), Florida (Lauderhill muck) and Iowa (Canisteo). Air-dried samples were ground to pass a 2-mm sieve. The soils were characterized for pH, particle size fractions (Gee and Bauder 1986), and total carbon (C) and nitrogen (N) (Vario Max-ELEMENTAR CN-analyzer D63452 Hanau; Germany).

**Table 1:** Selected properties of the four soils used in this study to assay for maltase activity

Soil	Classification	pH	Total C (g kg^−1^)	Total N (g kg^−1^)	Sand (g kg^−1^)	Clay (g kg^−1^)
Decatur	Rhodic paleudult	5.2	10.0	1.1	150	270
Canisteo	Typic haplaquoll	6.0	27.4	2.6	210	450
Houston	Typic chlomodert	7.2	48.2	4.2	250	490
Lauderhill muck	Lithic haplosaprists	7.3	242.5	17.2	33	260

### Reagents

*Toluene*: Fisher certified reagent.

*Maltose solution* (50 mM): Prepared by dissolving 18 g maltose monohydrate (Sigma Chemical Co., St. Louis, MO) in about 800 mL of sodium acetate buffer (67 mM, pH 5.0) and adjusting the volume to 1 L with the same buffer and stored at 4 °C.

*Sodium acetate buffer* (67 mM, pH 5.0): Prepared by dissolving 9.1 g sodium acetate trihydrate crystals (Sigma Chemical Co., St. Louis, MO) in about 700 mL of deionized water. The mixture is titrated to pH 5.0 with 99% glacial acetic acid and volume adjusted to 1 L with deionized water.

*Sulfuric acid* (12 N): Prepared by diluting 333.33 mL of 36 N sulfuric acid in 600 mL of deionized water in a 1 L volumetric flask and adjusting the volume with deionized water.

*Glucose standard*: Glucose standard (Sigma Chemical Co., St. Louis, MO) was prepared based on the manufacturer’s technical bulletin instructions.

*Glucose oxidase–peroxidase*: Glucose oxidase–peroxidase enzyme (Sigma Chemical Co., St. Louis, MO) was prepared based on the manufacturer’s instructions and stored at 4 °C in an opaque bottle wrapped with aluminum foil. The reagent is stable for up to 3 months.

### Enzyme assay

Unless indicated otherwise, soil maltase activity was assayed by placing 1.0 g of soil (air-dried, <2 mm) into a 50 mL plastic centrifuge tube, and then treated with 0.2 mL toluene and 4.3 mL sodium acetate buffer (67 mM, pH 5.0). The tube was swirled for a few seconds to mix the contents and 0.5 mL of 50 mM maltose (prepared in sodium acetate buffer) added to obtain a 5 mM maltose concentration in a 6 mL final volume. The centrifuge tube was stoppered, swirled for a few seconds and incubated at 37 °C for 1 h. After incubation, the tube was placed in a boiling water bath (98 °C) on a hot plate for 5 min to stop the enzyme activity. The tube was then removed, cooled and the soil suspension centrifuged at 8,000*g* for 10 min. The supernatant was filtered through a 0.45 μm cellulose acetate filter into a 50 mL tube (Corning Inc., Corning NY). One mL of filtrate aliquot was placed in another labeled tube and placed in a water bath at 37 °C followed by the addition of 2 mL glucose oxidase–peroxidase, thoroughly mixed and incubated for exactly 30 min. The reaction was stopped by adding 2 mL of 12 N H_2_SO_4_ and mixed thoroughly.

**Fig. 1:**
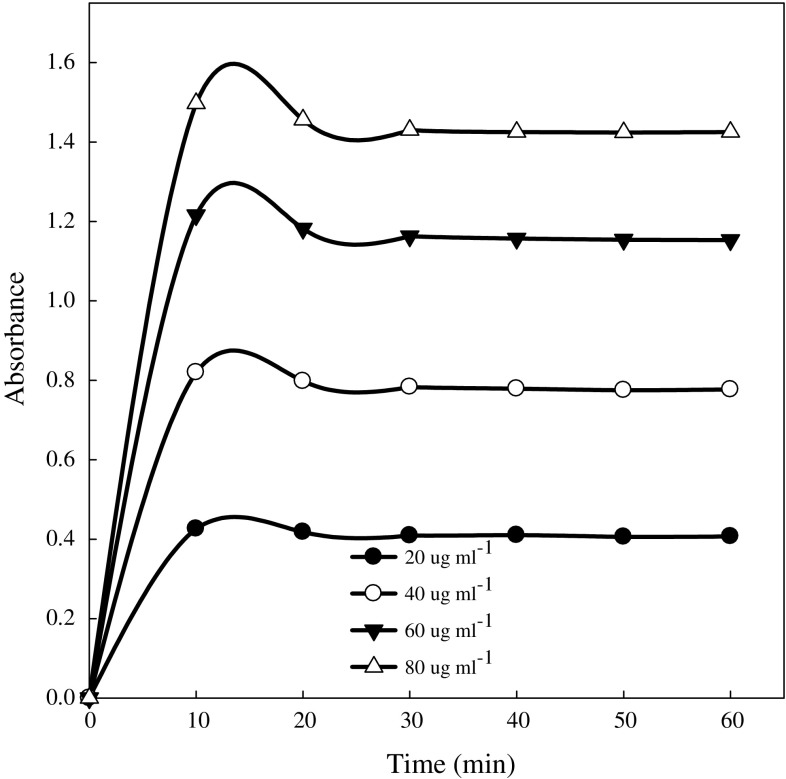
Calibration graphs of glucose standards

Glucose standard solutions was prepared using a ready-to-use d-glucose (product 22 code G 3285) from Sigma-Aldrich Co and the standard curve (Fig. 1) developed using 20, 40, 60, and 80 μg mL^−1^ glucose. The standards were subjected to the same reaction conditions as the soil filtrate described above. Control experiments were included with each soil sample to account for the glucose from sources other than maltase activity. The control consisted of incubating soil containing 0.2 mL toluene and 4.3 mL sodium acetate buffer (67 mM, pH 5.0) at 37 °C for 1 h. Thereafter, 0.5 mL of buffered 50 mM maltose was added to the reaction mixture and subjected to the same standard procedure described above.

The pink color intensity was measured after 30 min at 540 nm against a sodium acetate reagent blank using a spectrophotometer (GENESYS 10 spectrophotometer, Model 335902P-000, Thermo Electron Corp., Madison, WI). To ensure that measurements were done when the pink color was most stable, standards were measured at various time intervals between 10 and 60 min. Tests indicated that the pink color is most stable under laboratory lighting conditions after 30 min. The method used gives quantitative (ca. 100%) recovery of reducing sugars added to soils.

### Kinetic determination

The kinetic parameters were assessed by estimating maltase activity at different maltose concentrations (0.0, 0.5, 2.0, 6.0, 10 and 14 mM). The *K*_m_ and *V*_max_ values were calculated using both linear and non-linear transformations of the Michaelis–Menten equation. The measurements to determine the temperature coefficients (*Q*_10_) were at 10 °C intervals (between 0 and 80 °C), and the activation energy (*E*_a_), was determined using the Arrhenius equation.

## Results and discussion

### Effect of buffered pH

**Fig. 2:**
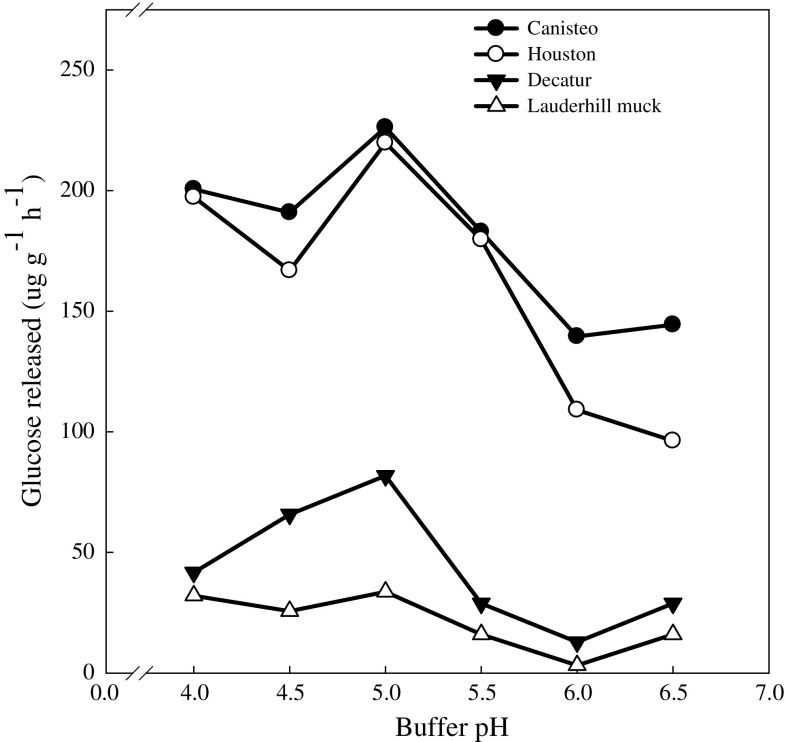
Soil maltase activity at various pH

Enzymes are sensitive to pH of the reaction medium. The intensity of their excretion by plant roots and microorganisms is determined by their requirements for substrates affected by soil pH. To ascertain that soil maltase activity was assayed at its optimum pH, we used buffered pH (in order to avoid the effect of ionic strength and test only the effects of pH) values ranging from 4.0 to 6.5 (Fig. 2). The effect of pH on maltase activity indicates that the optimum pH was 5.0. The optimum pH reported for maltase from microbes isolated from soils has ranged from 4.0 (Gomes et al. 2005) to 6.6 (McWethy and Hartman 1979). Eivazi and Tabatabai (1988), using *p*-nitrophenyl α-d-glucopyranoside as substrate in modified universal buffer (MUB) reported a pH optimum at 6.0. The optimum pH value (5.0) obtained in this study is close to the optima pH of other reported enzymes involved in C mineralization in soils (Eivazi and Tabatabai 1988; Deng and Tabatabai 1994).

Various buffers have been used to assay for maltase activity from microbes isolated from soils and soil extracts. These include phosphate, citrate, succinate, and acetate. Among these buffers, the phosphate buffers have been the most used with isolates (Berthelot and Delmotte 1999; Suzuki et al. 1976; Wang and Hartman 1976). Recent studies, however; have used the acetate buffer (Gomes et al. 2005; Kobayashi et al. 2003). In this study, various buffer strengths were used including 50, 67 and 100 mM. These buffer strengths when tested for soil maltase activity (data not shown) showed that 67 mM buffer strength produced the highest activity. Test with potassium phosphate buffer showed that it extracts humic materials from high organic matter soils (Al-Turki and Dick 2003).

### Substrate concentration and amount of soil

**Fig. 3:**
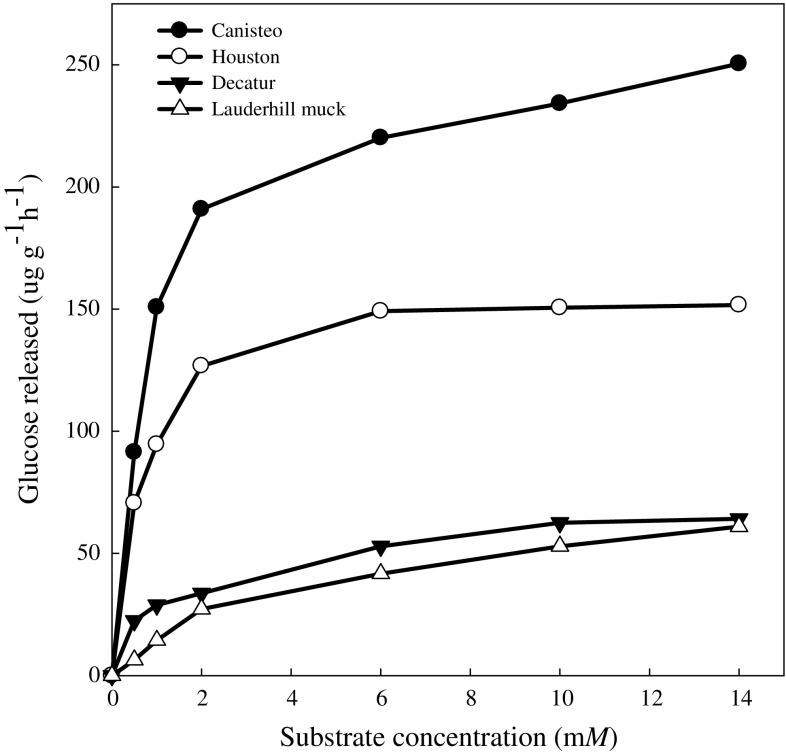
Soil maltase activity at various substrate concentrations

**Fig. 4:**
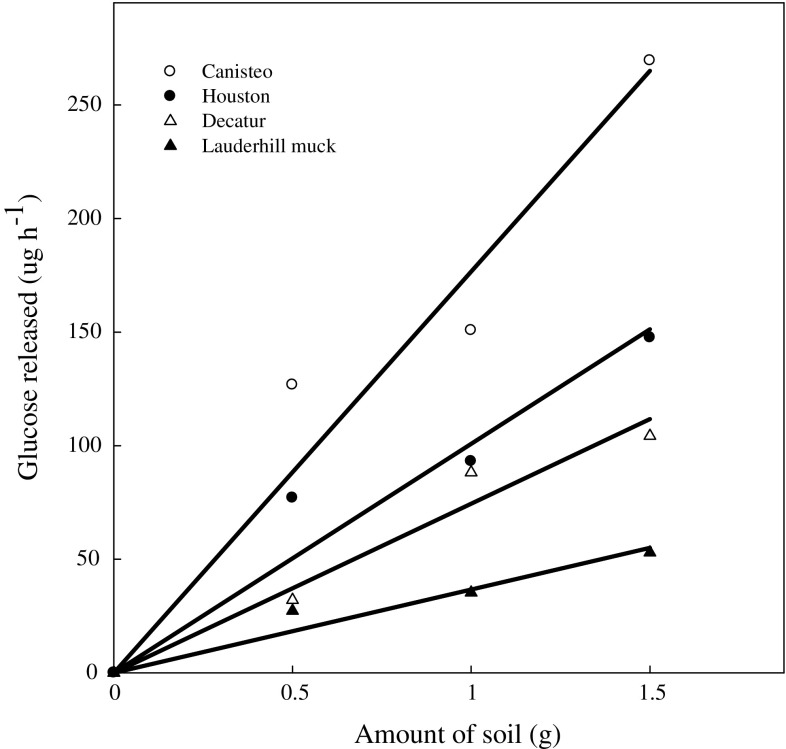
Soil maltase activity in different amounts of soil

Results obtained from varying the substrate concentrations used in this study followed the patterns reported for classical theory of enzyme kinetics (Fig. 3). The data obtained showed that we could assay the activity using maltose concentrations ranging from 5 to 8 mM. Various maltose concentrations including 3.3 mM (Wang and Hartman 1976), 5 mM (Bailey and Howard 1963), and 0.5% w/v (Wimmer et al. 1997) have been used to assay maltase activity. A linear relationship between the amount of soil and the amount of glucose released (Fig. 4), suggest 1.0 g soil was satisfactory at the maltose concentration of 5 mM, which is about three times the average *K*_m_ values for all the soils used in this study.

### Time and temperature of incubation

**Fig. 5:**
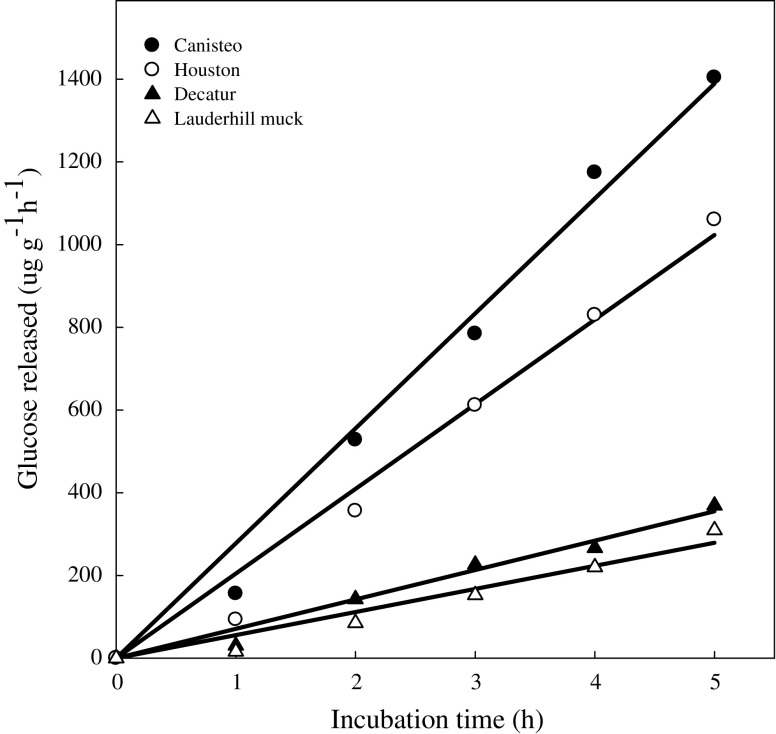
Soil maltase activity at various incubation times

**Fig. 6:**
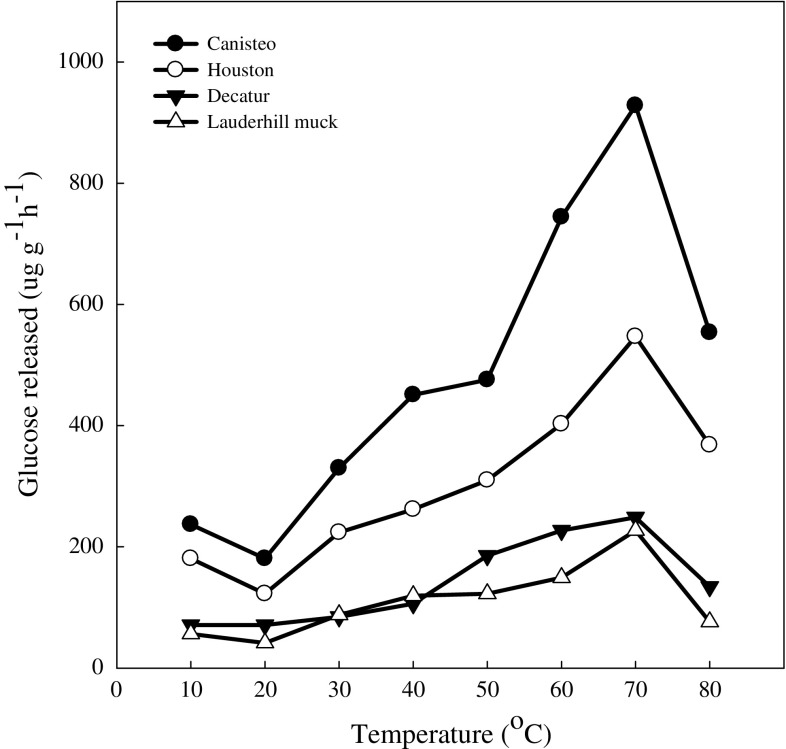
Soil maltase activity at various incubation temperatures

Evaluation of the effect of incubation time on the activity of this enzyme using the adopted method revealed a linear relationship up to 5 h in all the soils used in this study (Fig. 5. The observed linear relationship indicates that the 1 h incubation time used was not constrained by microbial utilization of the glucose formed, nor limited by enzyme stability or availability of the substrate. Skujins (1976) suggested that an assay for soil enzymes should be limited to short incubation time to reduce risks or errors from increased microbial activity with lengthy incubation time. Decreasing activity during incubation occurred at 70 °C (Fig. 6) suggesting the enzyme in soil is fairly stable. The optimum temperature for maltase extracted from soil microbes has been reported at 45 °C (Wang and Hartman 1976), 65 °C (Kobayashi et al. 2003), 70 °C (Gomes et al. 2005), and 75 °C (Wimmer et al. 1997).

The temperature required to denature an enzyme without soil is about 10–15 °C lower than that required to inactivate the same enzyme in the presence of soils (Skujins 1976). Soil maltase activity was assayed at 37 °C because mesophilic and thermophilic microbes in soils are reported active at this temperature (Wang and Hartman 1976; Bailey and Howard 1963; Eivazi and Tabatabai 1988). This temperature is consistent with the temperature used in most published procedures for determining other enzyme activities in several biological materials, including soils (Tabatabai 1994; Senwo and Tabatabai 1996). Although the inactivation temperature is higher than that of aspartase (Senwo and Tabatabai 1996) it is similar to those reported for arylsulfatase, rhodanase, phosphodiesterase, and amidase activities (Frankenberger and Tabatabai 1980).

### Activation energy and kinetic parameters

**Fig. 7:**
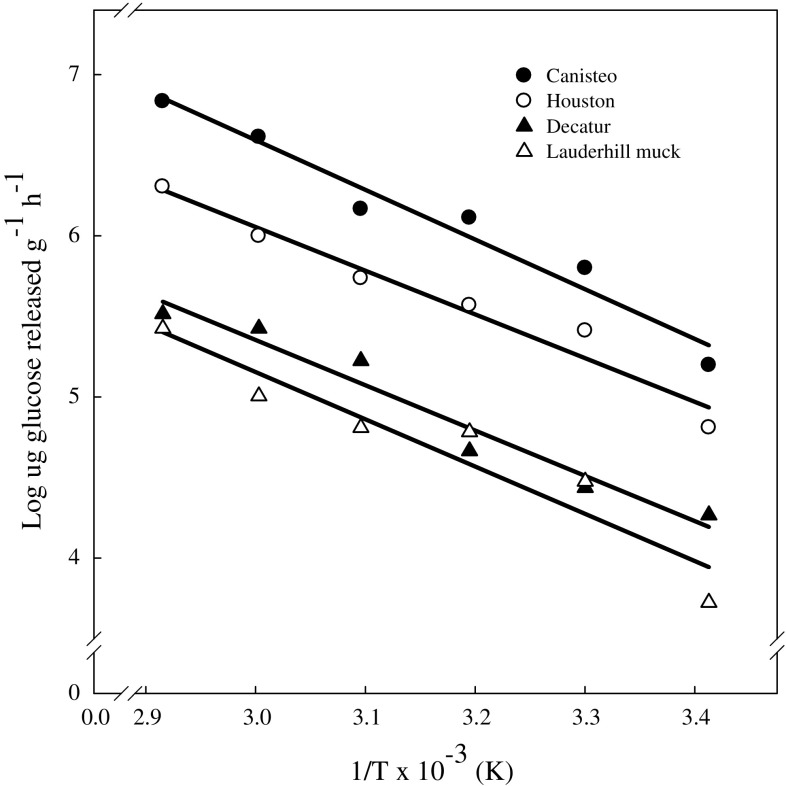
Arrhenius equation plot of soil maltase activity values

When an enzyme reaction obeys the Arrhenius equation [*k* = *A.* exp (−*E*_a_/*RT*)], the activation energy (*E*_a_) can be estimated from the logarithmic transformed equation [log *k* = (−*E*_a_/2.303*RT*) + log *A*]. Levine (1988) suggested the *E*_a_ value is approximately equal to the difference in energy between the reactants and the transition state. In this study, the slope of the Arrhenius equation plots for soils (Fig. 7) was linear between 20 and 70 °C suggesting similar measurements in enzyme activity. The calculated *E*_a_ ranged between 34.1 kJ mol^−1^ (Houston soil) to 57.2 kJ mol^−1^ (Canisteo soil) with an average value of 42.0 kJ mol^−1^ (Table 2). Eivazi and Tabatabai (1988) reported an average *E*_a_ for α-glucosidase activity in soils to be 43.1 kJ mol^−1^ when *p*-nitrophenyl α-d-glucopyranoside was used as the substrate. The values reported in this study are within the ranges reported for other soil enzymes (Tabatabai 1994).

**Table 2:** Activation energies (*E*_a_) and average temperature quotient (*Q*_10_) of soil maltase activity

Soil	*E*_a_ (kJ mol^−1^)	Mean *Q*_10_
Decatur	36.6	1.6
Canisteo	57.2	1.7
Houston	34.1	1.5
Lauderhill muck	40.0	1.9

$$ Q_{10} = \frac{{{\text{Maltase\,activity\,at }}T(^{\circ } {\text{C}})}}{{{\text{Maltase\,activity\,at }}T(^{\circ} {\text{C}}) - 10\,{^{\circ }}{\text{C}}}} $$

**Fig. 8:**
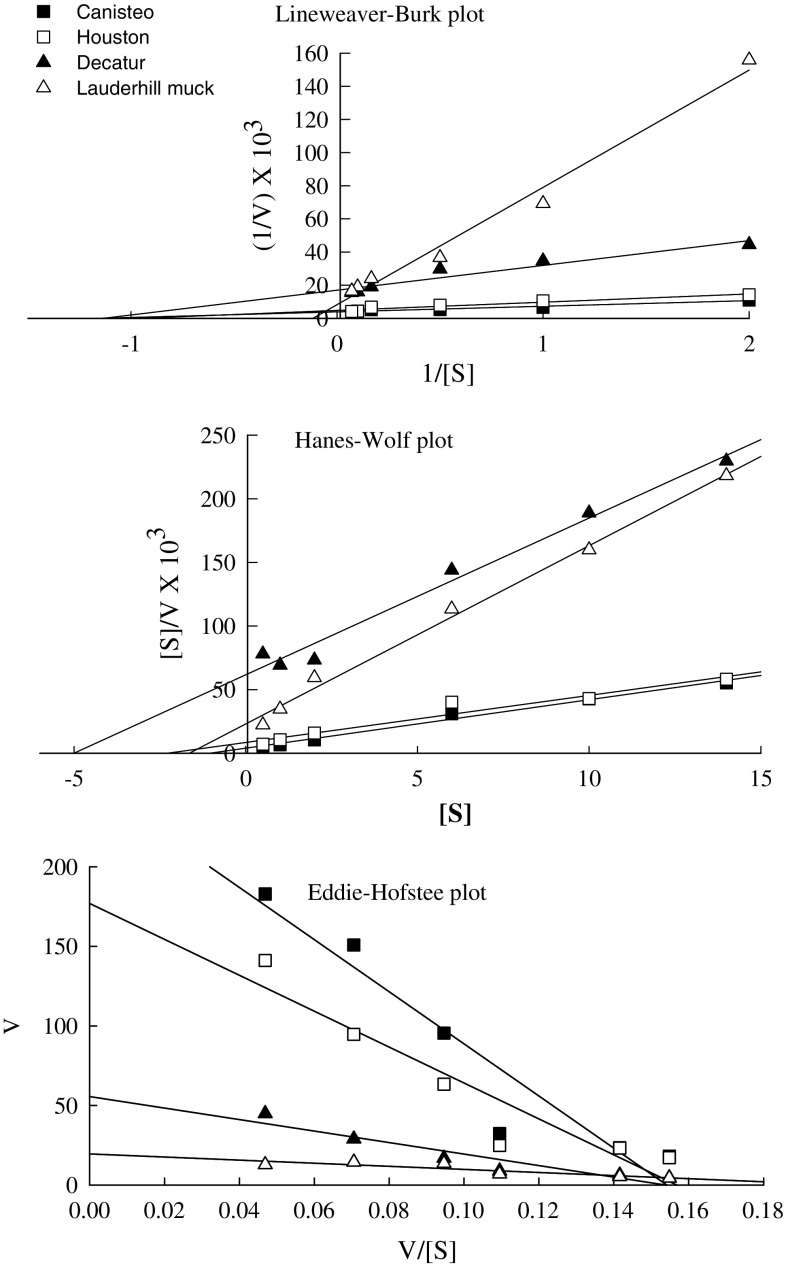
Transformation plots of Michaelis–Menten equation for soil maltase activity (*V* reaction velocity, *S* substrate concentration)

The estimated *Q*_10_ values for the activity ranged from 1.5 (Houston soil) to 1.9 (Lauderhill muck soil) and averaged about 1.7 (Table 2) indicating that the reaction rates are almost doubled for every 10 °C increase in temperature. This probably indicates that a 1 °C difference in the reaction temperature may result in approximately 10% variation among analyses. The average *K*_m_ value using the linear transformations ranged from 0.8 to 6.5 mM, while *V*_max_ ranged from 59 to 270 μg g^−1^ h^−1^. The three transformations (Fig. 8) showed considerable variation because each transformation gives different weight to errors in the variables (Dowd and Riggs 1965). A non-linear regression fit was also used, which gave *K*_m_ values ranging from 0.8 to 4.4 mM, and *V*_max_ values ranging from 70 to 257 μg g^−1^ h^−1^ (Table 3). 

The differences in *K*_m_ and *V*_max_ values for the soils tested are likely due to the presence of different soil enzyme concentrations from various sources. Kobayashi et al. (2003) reported the *V*_max_ and *K*_m_ values for *Paecilomyces lilacinus* (fungus) maltase extracted from soil as 51.3 mM min^−1^ and 0.16 mM respectively. Other *K*_m_ values reported are 5.0 mM for *Bacillus subtilis* (Wang and Hartman 1976), 5.8 mM for *B*. *brevis* (McWethy and Hartman 1979), and 0.83 mM (Hutson and Manners 1965) from Alfalfa extracts. The *K*_m_ values of soil maltase activity determined in this study, compared to those of enzymes purified from microorganisms. The standard deviation values of the activity ranged from 1.9 to 9.8 and the coefficient of variation (CV) determined for the proposed method using the soils studied was <6.0% (Table 4).

**Table 3:** *K*_m_ and *V*_max_ values of soil maltase activity determined by proposed method

Soil	*K*_m_ (mM)		*V*_max_ (μg glucose g^−1^h^−1^)	
Lineweaver–Burk plot (1/*V* vs. 1/[*S*])				
Decatur	0.9	1.6^a^	59	70^b^
Canisteo	0.9	0.8	256	250
Houston	1.0	2.0	208	257
Lauderhill muck	6.5	4.4	94	78
Average	2.3		154	
Hanes-Wolf plot (*V* vs. V/[*S*])				
Decatur	1.6		71	
Canisteo	1.0		263	
Houston	2.0		270	
Lauderhill muck	4.2		68	
Average	2.2		168	
Eddie–Hofstee plot ([*S*] vs. [*S*]/*V*)				
Decatur	1.0		63	
Canisteo	0.8		251	
Houston	1.0		229	
Lauderhill muck	3.7		64	
Average	1.6		152	

^a^*K*_m_ of non-linear regression fit for each soil

^b^*V*_max_ of non-linear regression fit for each soil

**Table 4:** SD and CV determined by proposed method

Soil	Maltase activity			
	Range (μg glucose g^−1^ soil h^−1^)	Mean^a^ (μg glucose g^−1^ soil h^−1^)	SD^b^ (μg glucose g^−1^ soil h^−1^)	CV^c^ (μg glucose g^−1^ soil h^−1^)
Decatur	51–54	52	2.4	4.6
Canisteo	180–200	186	9.8	5.3
Houston	143–149	145	2.3	1.6
Lauderhill muck	43–48	45	1.9	4.3

^a^Mean of six replications

^b^Standard deviation

^c^Coefficient of variation

## Conclusion

While we recognize the availability of established assay protocols to determine the activity of α-glucosidase (referred in other literature as maltase) based on the *p*-nitrophenol (artificial product) released from *p*-nitrophenyl-α-d-glucopyranoside (artificial substrate), our interest was to assay its activity by determining the glucose (natural product) released from maltose (natural substrate). With the promotion of organic agriculture and the use of poultry litter as soil amendments, which results in the addition of maltose into the soil system, it is worth developing alternative assay protocols for the determination of maltase activity using natural substrate (maltose) and measurement of its natural product (glucose). The assay protocol proposed for determining soil maltase using maltose as substrate and the released glucose using a glucose oxidase–peroxidase system has not been explored or investigated to the best of our knowledge. The proposed assay protocol is sensitive and detects low glucose levels of 0.004 mg glucose per g of soil.
